# From lymphatic endothelial cell migration to formation of tubular lymphatic vascular network

**DOI:** 10.3389/fphys.2023.1124696

**Published:** 2023-02-21

**Authors:** Tomohiro Shiiya, Masanori Hirashima

**Affiliations:** Division of Pharmacology, Graduate School of Medical and Dental Sciences, Niigata University, Niigata, Japan

**Keywords:** cell adhesion, cell polarization, chemotactic factor, lymphatic endothelial cell, migration

## Abstract

During development, lymphatic endothelial cell (LEC) progenitors differentiate from venous endothelial cells only in limited regions of the body. Thus, LEC migration and subsequent tube formation are essential processes for the development of tubular lymphatic vascular network throughout the body. In this review, we discuss chemotactic factors, LEC-extracellular matrix interactions and planar cell polarity regulating LEC migration and formation of tubular lymphatic vessels. Insights into molecular mechanisms underlying these processes will help in understanding not only physiological lymphatic vascular development but lymphangiogenesis associated with pathological conditions such as tumors and inflammation.

## Introduction

The lymphatic system plays pivotal roles in fluid homeostasis, immune cell trafficking and dietary lipid uptake. Lymphatic vascular dysfunction leads to lymphedema, disturbed immune responses and lipid malabsorption. The lymphatic vasculature develops primarily from pre-existing veins, while non-venous derived lymphatic endothelial cells (LECs) have been reported in specific tissues (Oliver et al., 2020). The differentiation of LEC progenitors depends on expression of the transcription factor SRY-related HMG-box 18 (Sox18) and the transcription factor prospero-related homeobox gene 1 (Prox1) (Wigle and Oliver, 1999; Francois et al., 2008). Prox1 maintains the expression of receptor tyrosine kinase of vascular endothelial growth factor receptor 3 (VEGFR3), while VEGFR3 signaling upregulates Prox1 expression. This feedback loop between these two key players results in the lineage and maintenance of LECs (Srinivasan et al., 2014). The differentiated LECs subsequently proliferate and migrate away from the vein to establish tubular lymphatic vascular network throughout the body. LEC migration is induced by chemotactic factors such as VEGF-C and modulated by the interactions with extracellular matrix (ECM). Furthermore, the regulation of planar cell polarity (PCP) has been implicated in the directed migration of LECs and formation of tubular structure. In this review, we discuss the molecular mechanisms underlying these processes from LEC migration to formation of tubular lymphatic vascular network.

### Chemotactic factors

**FIGURE 1:**
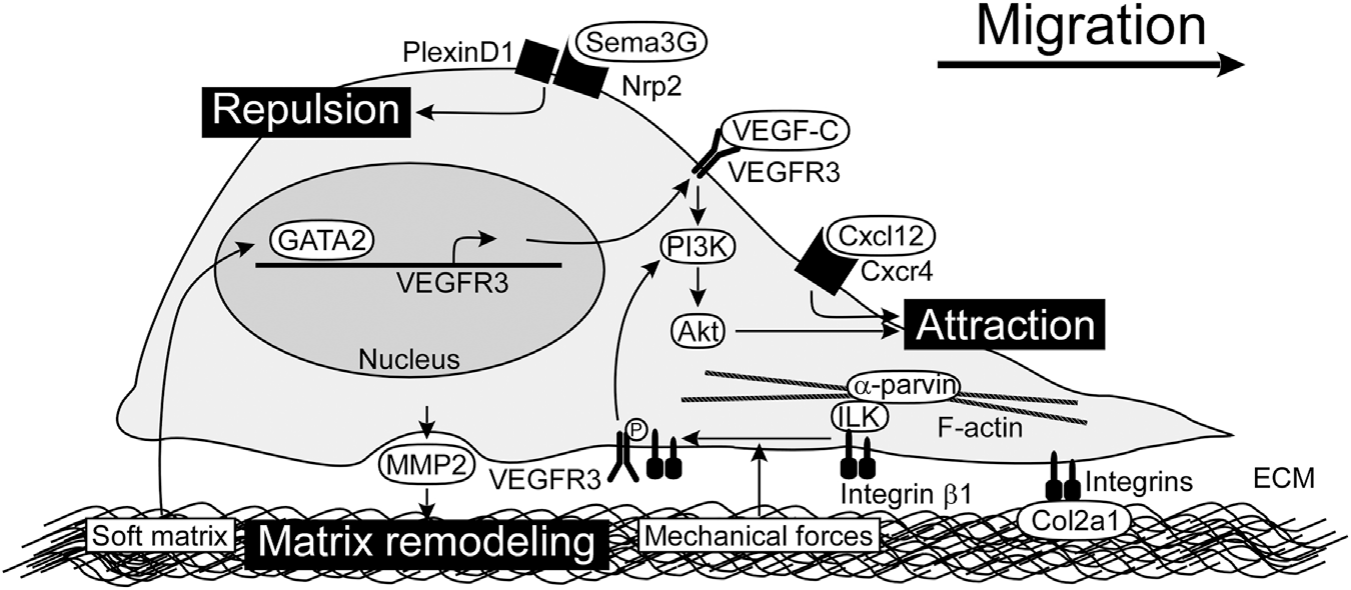
Chemotactic factors and LEC-ECM interactions for LEC migration. VEGF-C/VEGFR3/PI3K/Akt pathway is the main signaling for LEC migration. Cxcl12 also promotes LEC migration. Sema3G serves as a repulsive factor which acts through Nrp2/PlexinD1 receptor complex. Integrins expressed by LECs play a crucial role in adhesion to ECM as well as modulation of VEGFR3 signals. By sensing mechanical forces, integrin β1 is released from ILK and interacts with VEGFR3. MMP2 produced by LECs perform matrix remodeling, possibly modulating matrix stiffness. Soft matrix induces GATA2 expression, which enhances VEGFR3 expression. ECM, extracellular matrix; ILK, integrin-linked kinase; LEC, lymphatic endothelial cell; MMP2, matrix metalloproteinase 2; Nrp2, neuropilin-2; PI3K, phosphoinositide 3-kinase; Sema3G, Semaphorin 3G; VEGF-C, vascular endothelial growth factor C; VEGFR3, vascular endothelial growth factor receptor 3.

LECs originated from the jugular veins migrate under the control of chemotactic factors in embryos. Some factors promote LEC migration, while others provide a repulsive cue to LECs (Figure 1). These factors work in concert to regulate the distribution of lymphatic vessels.

VEGF-C plays an important role as a chemotactic factor in LEC migration (Karkkainen et al., 2004). During embryonic development, VEGF-C produced by mesenchymal cells is required for dorsolateral migration of LECs from the jugular vein in mice (Hagerling et al., 2013). VEGF-C is produced in a premature form and is cleaved to a mature form in collagen and calcium-binding epidermal growth factor domains 1 (CCBE1) and a disintegrin and metalloprotease with thrombospondin motifs-3 (ADAMTS3)-dependent manner (Jeltsch et al., 2014). Genetic ablation of either Ccbe1 or Adamts3 in mice results in defective lymphatic vascular development, which is most likely caused by improper activation of VEGF-C (Hagerling et al., 2013; Janssen et al., 2016). Loss-of-function mutations in the *ADAMTS3* gene have been identified in hereditary lymphedema patients (Brouillard et al., 2017). The mature form of VEGF-C binds to VEGFR3 (also known as Flt4) and activates intracellular signaling pathways, including phosphoinositide 3-kinase (PI3K)/Akt (also known as protein kinase B) and mitogen activated protein kinase (MAPK)/extracellular signal-regulated kinase (ERK). Neuropilin-2 (Nrp2) forms a complex with VEGFR3 to facilitate VEGF-C-mediated migration (Xu et al., 2010). Both Akt and ERK pathways are important for human dermal LEC migration *in vitro* (Deng et al., 2015), and PI3K/Akt pathway has especially been characterized as the regulator of lymphatic vascular morphogenesis *in vivo*. Somatic activating mutations in the *PIK3CA* gene encoding the p110α catalytic subunit of PI3K have been identified as causative genes of lymphatic malformations which are congenital developmental anomalies with variable size of fluid-filled lymphatic cysts (Makinen et al., 2021). Pik3ca mutant LECs show the spiky morphology of active sprouts in mouse embryos and migrate faster, compared with control LECs *in vitro* (Martinez-Corral et al., 2020). Activated PI3K phosphorylates phosphatidylinositol-4, 5-bisphosphate to create phosphatidylinositol-3, 4, 5-triphosphate, which activates Akt. This process is inhibited by phosphatase and tensin homolog (PTEN), and loss of PTEN enhances Akt activation. Actually, PTEN inhibits the PI3K/Akt pathway downstream of VEGF-C/VEGFR3 activation. Lymphatic-specific deletion of Pten in mice leads to the increased lymphatic vessel density, diameter, and branching (Kataru et al., 2021). Genetic ablation of Akt1 in mice leads to the reduced size of lymphatic capillaries and defects in the maturation of collecting lymphatic vessels and valve development (Zhou et al., 2010). These studies demonstrated that VEGF-C/VEGFR3/PI3K/Akt pathway plays a crucial role in LEC migration.

Chemokine ligands Cxcl12a and Cxcl12b induce LEC migration *via* Cxcr4a or Cxcr4b receptor. The chemokine signaling orchestrates the stepwise assembly and patterning of the trunk lymphatic network in zebrafish (Cha et al., 2012). It has been shown that Cxcl12a-mediated LEC migration are regulated by miR-126a which binds the 5′untranslated region of Cxcl12a mRNA and enhances the translation (Chen et al., 2016).

Due to predominant expression of VEGF-C and Cxcl12 in arteries, initial LEC migration instructed by these attractive factors is observed along arteries (Vaahtomeri et al., 2017). The guidance factor Semaphorin 3G (Sema3G) plays a key role in subsequent broad distribution of lymphatic vasculature in mouse embryonic skin. Sema3G produced by arterial endothelial cells binds to the LEC receptors Nrp2. Nrp2 binds to the coreceptor PlexinD1 and provides a repulsive cue to LECs during lymphatic vascular patterning. Genetic ablation of either Sema3G or PlexinD1 in mice results in the limited distribution of lymphatic vasculature near arteries (Liu et al., 2016).

### LEC-ECM interactions

LEC migration is based on the interactions between LECs and ECM. The ECM is composed of various structural proteins such as collagen, fibronectin, and hyaluronan. Cell anchorage to ECM creates a strong connection between the intracellular and extracellular environments (Giancotti and Ruoslahti, 1999). Regulators of LEC-ECM interactions are shown in Figure 1.

Integrin is the main component of ECM receptor complex in LECs and serve as a heterodimeric protein consisting of eighteen α and eight β subunits (Hynes, 2002). These receptors involve in cell motility by supporting adhesion to the ECM and by linking *via* adapters with actin cytoskeleton (Lock et al., 2008). In addition to a role in cell adhesion, integrins transduce signals by associating with adapter proteins that connect to the cytoskeleton and cytoplasmic kinases. As integrins bind to ECM, they become clustered in the plane of the cell membrane and associated with a cytoskeletal and signaling complex that promotes the assembly of actin filaments. Integrin β1, the component of integrin α1β1, α2β1, or α4β1 has been implicated in lymphangiogenesis (Hong et al., 2004; Garmy-Susini et al., 2010; Kumaravel et al., 2020). Antagonists of integrin α5β1 inhibits inflammatory lymphangiogenesis in cornea (Dietrich et al., 2007). Knockdown of integrin α9, the component of integrin α9β1, in human LECs suppresses proliferation, adhesion, migration, and tube formation (Altiok et al., 2015). Integrin β1 reportedly responds to mechanical forces and activates VEGFR3. Integrin β1 interacts with integrin-linked kinase (ILK) which binds to α-parvin and F-actin cytoskeleton. Upon an increase in interstitial fluid volume, integrin β1 is released from ILK and interacts with VEGFR3 (Urner et al., 2019).

Type II collagen (Col2a1) is an important ECM component for LEC migration. Col2a1 secretion is controlled by a pathway mediating endoplasmic reticulum (ER)-Golgi anterograde transport. Membrane-bound transcription factor peptidase site-1 (Mbtps1) is a serine protease which cleaves and activates several transcription factors. Mbtps1 activates the transcription factor cAMP responsive element binding protein 3-like 2 (Creb3l2), which induces the transcription of *Sec23a* gene (Saito et al., 2009). The main function of Sec23a is to concentrate fully-folded proteins into vesicles on the ER. In *mbtps1* or *sec23a* mutant zebrafish, the defective secretion of Col2a1 protein affects LEC migration, resulting in the defective lymphatic vasculature in the trunk (Chaudhury et al., 2020).

Matrix stiffness affects LEC-ECM interactions. Matrix remodeling by matrix metalloproteinase 2 (MMP2) activity plays a role in lymphangiogenesis (Detry et al., 2012). LECs grown on a soft matrix exhibit increased expression of the transcription factor *GATA2* and downstream target genes involved in LEC migration and lymphangiogenesis. Analyses of mouse models demonstrate a cell-autonomous function of GATA2 in regulating LEC responsiveness to VEGF-C *via* VEGFR3 upregulation and in controlling LEC migration and sprouting *in vivo* (Frye et al., 2018).

### Planar cell polarity

**FIGURE 2:**
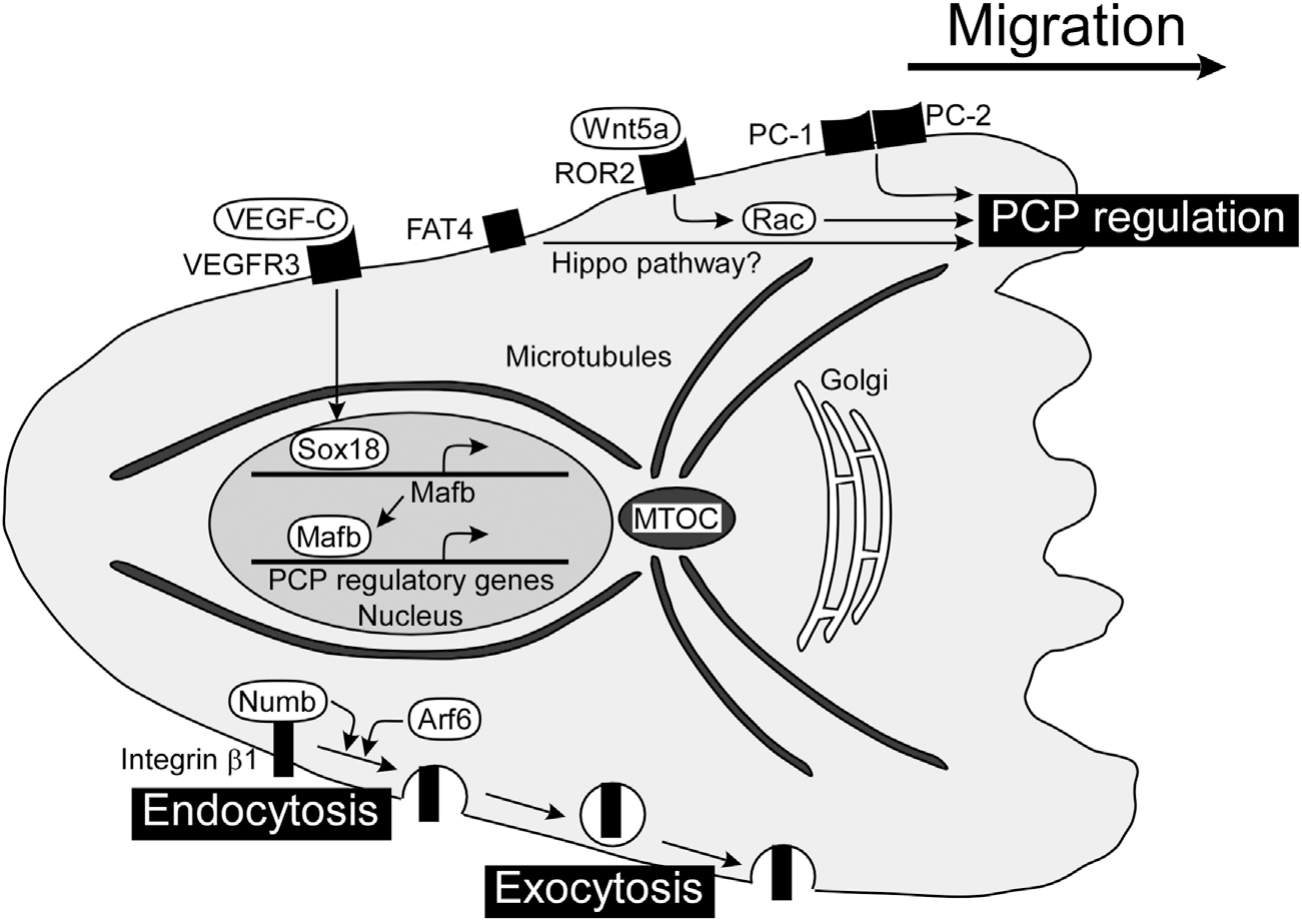
PCP regulators for directed migration of LECs and formation of tubular lymphatic vessels. PCP is established in a migrating cell with the localization of the MTOC and Golgi apparatus in front of the nucleus. There are several players regulating PCP in LECs, including Wnt5a, PCs, and FAT4, although the mechanisms of molecular interactions are not clear yet. VEGF-C/VEGFR3-dependent expression of Mafb induces gene expression involved in PCP regulation. The internalization and transport of integrin β1 towards the cell front enhance the establishment of front-rear polarity. FAT4, FAT tumor suppressor homolog 4; LEC, lymphatic endothelial cell; Mafb, MAF bZIP transcription factor B; MTOC, microtubule-organizing center; PC, polycystin; PCP, planar cell polarity; VEGF-C, vascular endothelial growth factor C; VEGFR3, vascular endothelial growth factor receptor 3; Wnt5a, wingless-type MMTV integration site family member 5a.

The migrating cell is highly polarized with complex regulatory pathways that spatially and temporally integrate its component processes. Establishing and maintaining PCP in response to extracellular stimuli appear to be mediated by a set of interlinked positive feedback loops (Ridley et al., 2003). PCP is a polarity axis organizing cells in the plane of the tissue and is essential for proper development and tissue homeostasis. It has been shown that PCP is related to directed migration of LECs and formation of tubular lymphatic vessels. Cell polarity is associated with reorientation of the microtubule-organizing center (MTOC) and the Golgi apparatus toward the cell front, leading to growth of microtubules and delivery of vesicles and proteins into this region (Ravichandran et al., 2020). PCP regulators in LECs are shown in Figure 2.

The Wingless-type MMTV integration site (WNT) family regulates numerous aspects of embryonic development including angiogenesis. WNTs constitute a large group of secreted lipid-modified signaling glycoproteins, and several receptors and co-receptors are involved in β-catenin-dependent canonical or non-canonical WNT signaling pathways. Wnt5a has been implicated in the formation of tubular lymphatic vessels by regulating the WNT/PCP pathway, since cyst-like lymphatics are detected in the skin of Wnt5a-deficient mice (Lutze et al., 2019). Wnt5a pathway is mediated *via* Rac GTPases, and Rac1 plays an important role in directed migration of LECs from the jugular vein in mouse embryos (D'Amico et al., 2009).

Polycystin (PC) is a cell surface receptor involved in cell-cell and cell-matrix interactions and is encoded by *PKD1* and *PKD2*, the genes responsible for autosomal dominant polycystic kidney disease (Harris and Torres, 2009). PC-1 is a large membrane receptor with a short intracellular C-terminus that binds to PC-2, which has homology to the transient receptor potential family of calcium-permeable cation channels (Gallagher et al., 2010). PC-1 and PC-2 act as important regulators of directed migration of LECs, and decreased vascular branching with cyst-like lymphatics is detected in *Pkd1*-or *Pkd2*-deficient mice (Coxam et al., 2014; Outeda et al., 2014).

The small GTPase Arf6 plays pivotal roles in a wide variety of cellular events such as endocytosis, exocytosis, and actin cytoskeleton reorganization. Arf6 regulates VEGF-C-dependent directed migration by enhancing the internalization of cell surface integrin β1 (Lin et al., 2017). Integrins internalized into vesicles *via* endocytosis at the cell rear are transported toward the cell front and used for new adhesions (Paul et al., 2015). Besides Arf6, the endocytic adaptor protein Numb binds integrin β1 and controls endocytosis of integrin for directed migration with the PAR complex composed of PAR-3, PAR-6 and atypical protein kinase C (Nishimura and Kaibuchi, 2007). The PAR complex controls the spatiotemporal dynamics of actin filaments and MTOC in directionally migrating cells (Crespo et al., 2014).

MAF bZIP transcription factor B (Mafb) has been identified as a downstream transcriptional effector of the VEGF-C/VEGFR3 axis, regulating the transcription factors PROX1, SOX18, COUP-TFII, and the kruppel-like transcription factor 4 in LECs (Dieterich et al., 2015; Koltowska et al., 2015). Mafb regulates morphogenesis of a subset of lymphatic beds, including the skin and diaphragm in mice (Dieterich et al., 2020; Rondon-Galeano et al., 2020). Mafba and Mafbb, two paralogs in zebrafish, tune the directionality of LEC migration in facial lymphatic development, without affecting cell motility (Arnold et al., 2022).

FAT tumor suppressor homolog 4 (FAT4) is atypical cadherin which has been implicated in Hippo pathway. FAT4 plays a role in polarization of LECs since cyst-like lymphatics are detected in Fat4-deficient mouse embryos (Betterman et al., 2020). YAP (Yes-associated protein) and TAZ (transcriptional coactivator with PDZ-binding motif) are final effectors of Hippo pathway. Lymphatic-specific YAP/TAZ depletion in mice leads to mispatterning of growing lymphatic plexus with cyst-like lymphatics (Cho et al., 2019). Apoptosis-stimulating protein of p53 (Aspp) 1 is an interacting protein with LATS kinases, another Hippo pathway-related molecule (Vigneron et al., 2010). Aspp regulates intercellular tension, and cyst-like lymphatics are detected in Aspp1-deficient mouse embryos (Hirashima et al., 2008; Matsuzawa et al., 2021). These results suggests that Hippo pathway regulates PCP of LECs, although the detailed mechanisms remain to be elucidated.

The core PCP proteins Celsr1 and Vangl2 reportedly regulate directed cell rearrangements during lymphatic valve formation by controlling the stabilization of endothelial adherens junctions (Tatin et al., 2013).

## Conclusion

In this review, we described molecular and cellular mechanisms of LEC migration and tube formation during lymphatic vascular development. Chemotactic factors and LEC-ECM interactions are important regulators of LEC migration and eventually to determine the distribution and morphogenesis of lymphatic vessels. Regulation of these factors will contribute to develop treatment for pathological lymphangiogenesis such as those in tumors and inflammation. PCP is related to directed migration of LECs and formation of tubular lymphatic vessels since improper PCP disrupts network formation, resulting in cyst-like lymphatics. These pathological conditions are similar to lymphatic malformations caused by somatic mutations, and insights into PCP regulation may help in the development of their treatment. Recently, it has been reported that the flow is involved in the regulation of blood endothelial cell polarity (Barbacena et al., 2022; Yuge et al., 2022). It may be intriguing to determine whether similar mechanisms exist in the regulation of LEC polarity to control morphogenesis of lymphatic vessels. Taken together, LEC migration and tube formation are key processes during the formation of functional lymphatic vessels, and studies on them will contribute to lymphatic vascular biology in health and diseases.

## References

[B1] Altiok E. Ecoiffier T. Sessa R. Yuen D. Grimaldo S. Tran C. (2015). Integrin alpha-9 mediates lymphatic valve formation in corneal lymphangiogenesis. Invest. Ophthalmol. Vis. Sci. 56, 6313–6319. 10.1167/iovs.15-17509 26431485 PMC4911102

[B2] Arnold H. Panara V. Hussmann M. Filipek-Gorniok B. Skoczylas R. Ranefall P. (2022). Mafba and mafbb differentially regulate lymphatic endothelial cell migration in topographically distinct manners. Cell Rep. 39, 110982. 10.1016/j.celrep.2022.110982 35732122

[B3] Barbacena P. Dominguez-Cejudo M. Fonseca C. G. Gomez-Gonzalez M. Faure L. M. Zarkada G. (2022). Competition for endothelial cell polarity drives vascular morphogenesis in the mouse retina. Dev. Cell 57, 2321–2333 e9. 10.1016/j.devcel.2022.09.002 36220082 PMC9552591

[B4] Betterman K. L. Sutton D. L. Secker G. A. Kazenwadel J. Oszmiana A. Lim L. (2020). Atypical cadherin FAT4 orchestrates lymphatic endothelial cell polarity in response to flow. J. Clin. Invest. 130, 3315–3328. 10.1172/JCI99027 32182215 PMC7260025

[B5] Brouillard P. Dupont L. Helaers R. Coulie R. Tiller G. E. Peeden J. (2017). Loss of ADAMTS3 activity causes Hennekam lymphangiectasia-lymphedema syndrome 3. Hum. Mol. Genet. 26, 4095–4104. 10.1093/hmg/ddx297 28985353

[B6] Cha Y. R. Fujita M. Butler M. Isogai S. Kochhan E. Siekmann A. F. (2012). Chemokine signaling directs trunk lymphatic network formation along the preexisting blood vasculature. Dev. Cell 22, 824–836. 10.1016/j.devcel.2012.01.011 22516200 PMC4182014

[B7] Chaudhury S. Okuda K. S. Koltowska K. Lagendijk A. K. Paterson S. Baillie G. J. (2020). Localised Collagen2a1 secretion supports lymphatic endothelial cell migration in the zebrafish embryo. Development 147, dev190983. 10.1242/dev.190983 32839180

[B8] Chen J. Zhu R. F. Li F. F. Liang Y. L. Wang C. Qin Y. W. (2016). MicroRNA-126a directs lymphangiogenesis through interacting with chemokine and Flt4 signaling in zebrafish. Arterioscler. Thromb. Vasc. Biol. 36, 2381–2393. 10.1161/ATVBAHA.116.308120 27789478

[B9] Cho H. Kim J. Ahn J. H. Hong Y. K. Makinen T. Lim D. S. (2019). YAP and TAZ negatively regulate Prox1 during developmental and pathologic lymphangiogenesis. Circ. Res. 124, 225–242. 10.1161/CIRCRESAHA.118.313707 30582452

[B10] Coxam B. Sabine A. Bower N. I. Smith K. A. Pichol-Thievend C. Skoczylas R. (2014). Pkd1 regulates lymphatic vascular morphogenesis during development. Cell Rep. 7, 623–633. 10.1016/j.celrep.2014.03.063 24767999 PMC5005109

[B11] Crespo C. L. Vernieri C. Keller P. J. Garre M. Bender J. R. Wittbrodt J. (2014). The PAR complex controls the spatiotemporal dynamics of F-actin and the MTOC in directionally migrating leukocytes. J. Cell Sci. 127, 4381–4395. 10.1242/jcs.146217 25179599 PMC4197085

[B12] D'Amico G. Jones D. T. Nye E. Sapienza K. Ramjuan A. R. Reynolds L. E. (2009). Regulation of lymphatic-blood vessel separation by endothelial Rac1. Development 136, 4043–4053. 10.1242/dev.035014 19906871 PMC2778747

[B13] Deng Y. Zhang X. Simons M. (2015). Molecular controls of lymphatic VEGFR3 signaling. Arterioscler. Thromb. Vasc. Biol. 35, 421–429. 10.1161/ATVBAHA.114.304881 25524775 PMC4304921

[B14] Detry B. Erpicum C. Paupert J. Blacher S. Maillard C. Bruyere F. (2012). Matrix metalloproteinase-2 governs lymphatic vessel formation as an interstitial collagenase. Blood 119, 5048–5056. 10.1182/blood-2011-12-400267 22490679

[B15] Dieterich L. C. Klein S. Mathelier A. Sliwa-Primorac A. Ma Q. Hong Y. K. (2015). DeepCAGE transcriptomics reveal an important role of the transcription factor MAFB in the lymphatic endothelium. Cell Rep. 13, 1493–1504. 10.1016/j.celrep.2015.10.002 26549461

[B16] Dieterich L. C. Tacconi C. Menzi F. Proulx S. T. Kapaklikaya K. Hamada M. (2020). Lymphatic MAFB regulates vascular patterning during developmental and pathological lymphangiogenesis. Angiogenesis 23, 411–423. 10.1007/s10456-020-09721-1 32307629 PMC7311381

[B17] Dietrich T. Onderka J. Bock F. Kruse F. E. Vossmeyer D. Stragies R. (2007). Inhibition of inflammatory lymphangiogenesis by integrin alpha5 blockade. Am. J. Pathol. 171, 361–372. 10.2353/ajpath.2007.060896 17591980 PMC1941598

[B18] Francois M. Caprini A. Hosking B. Orsenigo F. Wilhelm D. Browne C. (2008). Sox18 induces development of the lymphatic vasculature in mice. Nature 456, 643–647. 10.1038/nature07391 18931657

[B19] Frye M. Taddei A. Dierkes C. Martinez-Corral I. Fielden M. Ortsater H. (2018). Matrix stiffness controls lymphatic vessel formation through regulation of a GATA2-dependent transcriptional program. Nat. Commun. 9, 1511. 10.1038/s41467-018-03959-6 29666442 PMC5904183

[B20] Gallagher A. R. Germino G. G. Somlo S. (2010). Molecular advances in autosomal dominant polycystic kidney disease. Adv. Chronic Kidney Dis. 17, 118–130. 10.1053/j.ackd.2010.01.002 20219615 PMC2837604

[B21] Garmy-Susini B. Avraamides C. J. Schmid M. C. Foubert P. Ellies L. G. Barnes L. (2010). Integrin alpha4beta1 signaling is required for lymphangiogenesis and tumor metastasis. Cancer Res. 70, 3042–3051. 10.1158/0008-5472.CAN-09-3761 20388801 PMC2856096

[B22] Giancotti F. G. Ruoslahti E. (1999). Integrin signaling. Science 285, 1028–1032. 10.1126/science.285.5430.1028 10446041

[B23] Hagerling R. Pollmann C. Andreas M. Schmidt C. Nurmi H. Adams R. H. (2013). A novel multistep mechanism for initial lymphangiogenesis in mouse embryos based on ultramicroscopy. EMBO J. 32, 629–644. 10.1038/emboj.2012.340 23299940 PMC3590982

[B24] Harris P. C. Torres V. E. (2009). Polycystic kidney disease. Annu. Rev. Med. 60, 321–337. 10.1146/annurev.med.60.101707.125712 18947299 PMC2834200

[B25] Hirashima M. Sano K. Morisada T. Murakami K. Rossant J. Suda T. (2008). Lymphatic vessel assembly is impaired in Aspp1-deficient mouse embryos. Dev. Biol. 316, 149–159. 10.1016/j.ydbio.2008.01.023 18304521

[B26] Hong Y. K. Lange-Asschenfeldt B. Velasco P. Hirakawa S. Kunstfeld R. Brown L. F. (2004). VEGF-A promotes tissue repair-associated lymphatic vessel formation via VEGFR-2 and the alpha1beta1 and alpha2beta1 integrins. FASEB J. 18, 1111–1113. 10.1096/fj.03-1179fje 15132990

[B27] Hynes R. O. (2002). Integrins: Bidirectional, allosteric signaling machines. Cell 110, 673–687. 10.1016/s0092-8674(02)00971-6 12297042

[B28] Janssen L. Dupont L. Bekhouche M. Noel A. Leduc C. Voz M. (2016). ADAMTS3 activity is mandatory for embryonic lymphangiogenesis and regulates placental angiogenesis. Angiogenesis 19, 53–65. 10.1007/s10456-015-9488-z 26446156 PMC4700087

[B29] Jeltsch M. Jha S. K. Tvorogov D. Anisimov A. Leppanen V. M. Holopainen T. (2014). CCBE1 enhances lymphangiogenesis via A disintegrin and metalloprotease with thrombospondin motifs-3-mediated vascular endothelial growth factor-C activation. Circulation 129, 1962–1971. 10.1161/CIRCULATIONAHA.113.002779 24552833

[B30] Karkkainen M. J. Haiko P. Sainio K. Partanen J. Taipale J. Petrova T. V. (2004). Vascular endothelial growth factor C is required for sprouting of the first lymphatic vessels from embryonic veins. Nat. Immunol. 5, 74–80. 10.1038/ni1013 14634646

[B31] Kataru R. P. Baik J. E. Park H. J. Ly C. L. Shin J. Schwartz N. (2021). Lymphatic-specific intracellular modulation of receptor tyrosine kinase signaling improves lymphatic growth and function. Sci. Signal 14, eabc0836. 10.1126/scisignal.abc0836 34376570 PMC8567054

[B32] Koltowska K. Paterson S. Bower N. I. Baillie G. J. Lagendijk A. K. Astin J. W. (2015). Mafba is a downstream transcriptional effector of Vegfc signaling essential for embryonic lymphangiogenesis in zebrafish. Genes Dev. 29, 1618–1630. 10.1101/gad.263210.115 26253536 PMC4536310

[B33] Kumaravel S. Abbey C. A. Bayless K. J. Chakraborty S. (2020). The β1-integrin plays a key role in LEC invasion in an optimized 3-D collagen matrix model. Am. J. Physiol. Cell Physiol. 319, C1045–C1058. 10.1152/ajpcell.00299.2020 33052069

[B34] Lin Y. C. Ohbayashi N. Hongu T. Katagiri N. Funakoshi Y. Lee H. (2017). Arf6 in lymphatic endothelial cells regulates lymphangiogenesis by controlling directional cell migration. Sci. Rep. 7, 11431. 10.1038/s41598-017-11240-x 28900118 PMC5595869

[B35] Liu X. Uemura A. Fukushima Y. Yoshida Y. Hirashima M. (2016). Semaphorin 3G provides a repulsive guidance cue to lymphatic endothelial cells via neuropilin-2/PlexinD1. Cell Rep. 17, 2299–2311. 10.1016/j.celrep.2016.11.008 27880905

[B36] Lock J. G. Wehrle-Haller B. Stromblad S. (2008). Cell-matrix adhesion complexes: Master control machinery of cell migration. Semin. Cancer Biol. 18, 65–76. 10.1016/j.semcancer.2007.10.001 18023204

[B37] Lutze G. Haarmann A. Demanou Toukam J. A. Buttler K. Wilting J. Becker J. (2019). Non-canonical WNT-signaling controls differentiation of lymphatics and extension lymphangiogenesis via RAC and JNK signaling. Sci. Rep. 9, 4739. 10.1038/s41598-019-41299-7 30894622 PMC6426866

[B38] Makinen T. Boon L. M. Vikkula M. Alitalo K. (2021). Lymphatic malformations: Genetics, mechanisms and therapeutic strategies. Circ. Res. 129, 136–154. 10.1161/CIRCRESAHA.121.318142 34166072

[B39] Martinez-Corral I. Zhang Y. Petkova M. Ortsater H. Sjoberg S. Castillo S. D. (2020). Blockade of VEGF-C signaling inhibits lymphatic malformations driven by oncogenic PIK3CA mutation. Nat. Commun. 11, 2869. 10.1038/s41467-020-16496-y 32513927 PMC7280302

[B40] Matsuzawa K. Ohga H. Shigetomi K. Shiiya T. Hirashima M. Ikenouchi J. (2021). MAGIs regulate aPKC to enable balanced distribution of intercellular tension for epithelial sheet homeostasis. Commun. Biol. 4, 337. 10.1038/s42003-021-01874-z 33712709 PMC7954791

[B41] Nishimura T. Kaibuchi K. (2007). Numb controls integrin endocytosis for directional cell migration with aPKC and PAR-3. Dev. Cell 13, 15–28. 10.1016/j.devcel.2007.05.003 17609107

[B42] Oliver G. Kipnis J. Randolph G. J. Harvey N. L. (2020). The lymphatic vasculature in the 21(st) century: Novel functional roles in homeostasis and disease. Cell 182, 270–296. 10.1016/j.cell.2020.06.039 32707093 PMC7392116

[B43] Outeda P. Huso D. L. Fisher S. A. Halushka M. K. Kim H. Qian F. (2014). Polycystin signaling is required for directed endothelial cell migration and lymphatic development. Cell Rep. 7, 634–644. 10.1016/j.celrep.2014.03.064 24767998 PMC4040350

[B44] Paul N. R. Jacquemet G. Caswell P. T. (2015). Endocytic trafficking of integrins in cell migration. Curr. Biol. 25, R1092–R1105. 10.1016/j.cub.2015.09.049 26583903

[B45] Ravichandran Y. Goud B. Manneville J. B. (2020). The Golgi apparatus and cell polarity: Roles of the cytoskeleton, the Golgi matrix, and Golgi membranes. Curr. Opin. Cell Biol. 62, 104–113. 10.1016/j.ceb.2019.10.003 31751898

[B46] Ridley A. J. Schwartz M. A. Burridge K. Firtel R. A. Ginsberg M. H. Borisy G. (2003). Cell migration: Integrating signals from front to back. Science 302, 1704–1709. 10.1126/science.1092053 14657486

[B47] Rondon-Galeano M. Skoczylas R. Bower N. I. Simons C. Gordon E. Francois M. (2020). MAFB modulates the maturation of lymphatic vascular networks in mice. Dev. Dyn. 249, 1201–1216. 10.1002/dvdy.209 32525258

[B48] Saito A. Hino S. Murakami T. Kanemoto S. Kondo S. Saitoh M. (2009). Regulation of endoplasmic reticulum stress response by a BBF2H7-mediated Sec23a pathway is essential for chondrogenesis. Nat. Cell Biol. 11, 1197–1204. 10.1038/ncb1962 19767744

[B49] Srinivasan R. S. Escobedo N. Yang Y. Interiano A. Dillard M. E. Finkelstein D. (2014). The Prox1-Vegfr3 feedback loop maintains the identity and the number of lymphatic endothelial cell progenitors. Genes Dev. 28, 2175–2187. 10.1101/gad.216226.113 25274728 PMC4180978

[B50] Tatin F. Taddei A. Weston A. Fuchs E. Devenport D. Tissir F. (2013). Planar cell polarity protein Celsr1 regulates endothelial adherens junctions and directed cell rearrangements during valve morphogenesis. Dev. Cell 26, 31–44. 10.1016/j.devcel.2013.05.015 23792146 PMC3714594

[B51] Urner S. Planas-Paz L. Hilger L. S. Henning C. Branopolski A. Kelly-Goss M. (2019). Identification of ILK as a critical regulator of VEGFR3 signalling and lymphatic vascular growth. EMBO J. 38, e99322. 10.15252/embj.201899322 30518533 PMC6331728

[B52] Vaahtomeri K. Karaman S. Makinen T. Alitalo K. (2017). Lymphangiogenesis guidance by paracrine and pericellular factors. Genes Dev. 31, 1615–1634. 10.1101/gad.303776.117 28947496 PMC5647933

[B53] Vigneron A. M. Ludwig R. L. Vousden K. H. (2010). Cytoplasmic ASPP1 inhibits apoptosis through the control of YAP. Genes Dev. 24, 2430–2439. 10.1101/gad.1954310 21041411 PMC2964753

[B54] Wigle J. T. Oliver G. (1999). Prox1 function is required for the development of the murine lymphatic system. Cell 98, 769–778. 10.1016/s0092-8674(00)81511-1 10499794

[B55] Xu Y. Yuan L. Mak J. Pardanaud L. Caunt M. Kasman I. (2010). Neuropilin-2 mediates VEGF-C-induced lymphatic sprouting together with VEGFR3. J. Cell Biol. 188, 115–130. 10.1083/jcb.200903137 20065093 PMC2812843

[B56] Yuge S. Nishiyama K. Arima Y. Hanada Y. Oguri-Nakamura E. Hanada S. (2022). Mechanical loading of intraluminal pressure mediates wound angiogenesis by regulating the TOCA family of F-BAR proteins. Nat. Commun. 13, 2594. 10.1038/s41467-022-30197-8 35551172 PMC9098626

[B57] Zhou F. Chang Z. Zhang L. Hong Y. K. Shen B. Wang B. (2010). Akt/Protein kinase B is required for lymphatic network formation, remodeling, and valve development. Am. J. Pathol. 177, 2124–2133. 10.2353/ajpath.2010.091301 20724596 PMC2947305

